# Tele-Impedance control of a virtual avatar based on EMG and M-IMU sensors: a proof-of-concept

**DOI:** 10.1038/s41598-024-68232-x

**Published:** 2024-08-09

**Authors:** Silvia Buscaglione, Alessia Noccaro, Nevio Tagliamonte, Giulia Ticchiarelli, Giovanni Di Pino, Domenico Formica

**Affiliations:** 1 NeXT Lab: Research Unit of Neurophysiology and Neuroengineering of Human-Technology Interaction, Universitá Campus Bio-Medico di Roma, 00128 Rome, Italy; 2 Neurorobotics Lab, Newcastle University, Newcastle upon Tyne, NE1 7RU UK; 3 CREO Lab: Research Unit of Advanced Robotics and Human-Centred Technologies, Universitá Campus Bio-Medico di Roma, 00128 Rome, Italy; 4 NeuroRobot Lab: Laboratory of Robotic Neurorehabilitation, Fondazione Santa Lucia, 00179 Rome, Italy

**Keywords:** Biomedical engineering, Motor control

## Abstract

The broad spread of cooperative robots into many application domains has resulted in a demand for intuitive and effective solutions for teleoperated control. A relevant role in teleoperation has been assumed by impedance controllers, that allow the increase of stability and accuracy during interaction. This paper aims to test a teleoperation method based on an impedance controller, namely tele-impedance control, that is usable in unstructured environments since it relies only on wearable sensors. The proposed solution maps the joint stiffness and position of the human user, computed through six EMG and two M-IMU sensors, into the remote system to be teleoperated. We developed a 2-DoFs virtual task involving virtual physical interactions to compare the performance of our solution with the one of a traditional position-based controller. The study has been conducted on five healthy participants, who experienced both controllers in two different sessions. The tele-impedance approach has proved to be less physically demanding and more intuitive than the position-based one. Experimental data also allow us to investigate the strategy employed by the volunteers in the case of remote interactions, while using the two controllers. Of note, even though with the position controller the variation of subject impedance has no effect on the virtual arm, participants still tend to regulate both impedance and position of their own arm.

## Introduction

The last decades have been characterized by a growing interest in teleoperated systems, i.e. devices controlled by means of a master interface and executing actions remotely. Thus, the main interest in teleoperation lies in its exploitation for the execution of actions in dangerous and hostile environments^1–5^. Especially due to the Covid-19 pandemic, the use of these systems has been pursued in several fields, such as rehabilitation, surgery, industry, and daily life^6,7^. Several position- or force- based control strategies have been implemented with the aim of reducing the instabilities in the case of interactions with the environment^8^; nonetheless, the performance of the developed controllers was still poor in the case of unpredictable interactions^9^. These difficulties led roboticists to look for a solution by taking inspiration from the field of neuroscience and motor control, i.e. by trying to develop strategies for managing interactions with human-like proficiency.

So far different human strategies have been pointed out, and in all of them the relevant role of the task-related impedance modulation was evidenced^10–14^. In particular, two main interaction strategies have been highlighted depending on the nature of the perturbation, i.e. stable and predictable or unstable and not predictable. In the first case, the central nervous system learns an *internal model* of the dynamics of the task, and utilizes it to compute the feedforward interaction force needed to compensate for the external perturbation^15^. Before the new internal model is learned, joint impedance is increased to guarantee stability during the interaction with the unknown perturbation, then, once the learning is completed, the impedance reverts back to lower values^16,17^. In the second case, when interactions are unstable, learning an internal model cannot help due to the unpredictability of the perturbing force field. Hence, humans can deal with unstable tasks solely with an increase of joint impedance, whose magnitude, shape and orientation are optimized with learning to reduce the muscular effort^13,18^.

Impedance regulation has been demonstrated in motor control studies to have an important role in increasing both the stability and accuracy of an interaction task. For this reason, it is often pursued also in robotics both through software^19^ or hardware^20^ solutions. In teleoperation tasks, a human-inspired impedance controller, namely *tele-impedance control*, was proposed in^9,21,22^ as a method to regulate both robot position and mechanical impedance. This control method maps both the estimated human position and stiffness on the remote system to be teleoperated. To this aim, the real-time computation of both human position and stiffness is required. Even if the former can be achieved in a straightforward way through well-known kinematic models^8^, the latter is more challenging since the mechanisms behind human impedance regulation are still not fully clarified and also non-linearities may introduce further complexity. The relevant role of muscular co-contraction for stiffness modulation^11,23,24^ guided the development of different models for impedance computation based on electromyography (EMG) signals. Some examples include the Hill-type muscle model^25–27^ or the model based on linear relations between muscular activation and joint stiffness or torques^11,12,28^.

The group of Ajoudani has provided some examples of tele-impedance controllers^9,29–31^. In these works, the endpoint stiffness (computed based on muscle activity) and position (computed by using an optoelectronic system) were combined to control a remote robot in operational space. However, despite the effectiveness of the approach, the camera-based solution adopted for position detection restricts the use of the proposed algorithm in structured environments. Moreover, the stiffness in the operational space could not be estimated with high accuracy in the whole workspace, due to its dependency on the arm configuration.

A possible solution to overcome these limitations is the design of a tele-impedance controller in joint space that involves only wearable sensors as the human body interface, i.e., EMG and Magneto-Inertial Measurement Units (M-IMUs), as we proposed in a previous study^32^. This solution is indeed suitable for use in unstructured environments, thus providing greater flexibility and opening up broader application opportunities. Additionally, since the proposed approach focuses on joint stiffness rather than task space stiffness, it reduces dependency on the arm’s configuration.

Other researchers exploited M-IMU and EMG sensors for tele-impedance control^33,34^. However, these solutions are either based on hand stiffness and not portable^33^, or they do not map human impedance to the robotic system, instead controlling the robot’s impedance based on its position in the workspace^34^.

In this paper, we aim to (1) verify the previously proposed portable tele-impedance control system in a real-time interaction task, (2) compare the proposed algorithm with a control mapping only the arm position, and (3) investigate human motor strategies adopted to manage interaction in the case of remote tasks, evaluating whether they rely on position, stiffness or both quantities to adapt to the force exerted on the environment. We analyzed these questions by assessing human performance in a teleoperation control task in virtual reality involving virtual physical interactions with the environment.

## Materials and methods

We set up a reaching task in virtual reality to test our tele-impedance control method by implementing a control torque composed of the compound information on the kinematic state and the viscoelastic properties of the human arm^32^. We used a planar task and asked the participants to execute arm movements in the horizontal plane (2-DoFs), thus involving the flexion-extension of the shoulder and the elbow.

**Figure 1:**
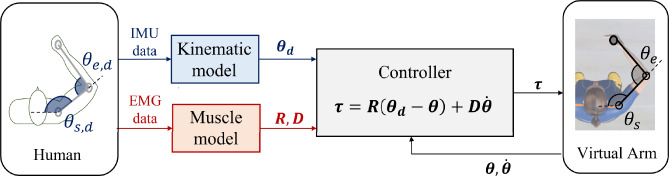
Block scheme of the proposed controller. The *Muscle model* computes the participant’s joint impedance in terms of stiffness (${\textbf{R}}$) and damping (${\textbf{D}}$) matrices, starting from pre-processed EMG data. Simultaneously, the participant’s joint angles, considered as desired set-points for the virtual arm to be teleoperated ($\pmb {\theta }_{d}$), are obtained through the *Kinematic model* based on quaternion data from M-IMU sensors. These outputs are sent to the controller for the computation of the control torque $\pmb {\tau }$, as in (3), together with the avatar arm kinematic state ($\pmb {\theta }$ and $\dot{\pmb {\theta }}$), which is retrieved from the engine of the virtual reality.

The data flow for the computation of the commanded torques is represented in Fig. 1A *Kinematic model* extracts the human’s joint angles vector $\pmb {\theta }_{d}$ using the M-IMU data. Simultaneously, a *Muscle model*^12^ derives the joint impedance, i.e. the matrices of joint stiffness (${\textbf{R}}$) and damping (${\textbf{D}}$), from EMG data recorded from the muscles indicated in Table 1. The output of both models is then used together with the actual joint angles ($\pmb {\theta }$) and velocities ($\dot{\pmb {\theta }}$) of the virtual arm to compute the torque vector $\pmb {\tau }$ for teleoperating the flexion-extension of the shoulder and elbow of the virtual arm, according to the Eq. (3).

The overall algorithm along with the two models have been previously detailed^32^. In "Muscle model and kinematic model" section we present a recap of the "Muscle model and Kinematic model" sections to facilitate the comprehension of the overall procedure. In "Control torque" section the torque computation is described, highlighting the difference between position (PC) and impedance (IC) controllers, which were both used by the participants for teleoperating the avatar in two different sessions. In "Experimental protocol" and Experimental setup" sections the details of the experimental protocol are presented, whereas "Performance metrics" and "Data analysis" sections report the evaluated metrics and the data elaboration process.

Hereafter, the subscripts *s* and *e* refer to shoulder and elbow joints respectively.

**Table 1:** Muscles considered for the computation of the arm stiffness the figure on the left represents a schematic illustration of the considered muscles with the respective sensors.

	*i*	Muscle	Typology
	1	Pectoralis major	S.M. Flexor
	2	Posterior deltoid	S.M. Extensor
	3	Brachioradilis	E.M. Flexor
	4	Lateral head of triceps brachii	E.M. Extensor
	5	Biceps brachii	B. Flexor
	6	Long head of triceps brachii	B. Extensor

The index *i* refers to the subscript of *u* in (1). For both shoulder (S) and elbow (E), one monoarticular (M) extensor (E) and flexor (F) were monitored together with biarticular (B) muscles.

### Muscle model and kinematic model

Even if the kinematic and muscle models used to implement the impedance control are described in detail in^32^, we recap here the main characteristics of the models so that the reader can better appreciate the experimental protocol and setup along with the data analysis.

#### Muscle model

The *Muscle Model* from^12^ was exploited to compute the joint stiffness matrix. Initially, a matrix of pseudo-stiffness $\varvec{\tilde{R}}=[\tilde{R}_{ss},\tilde{R}_{se}; \tilde{R}_{se},\tilde{R}_{ee}]$ was computed by using:

1 $$\begin{aligned} \tilde{R}_{{ss}} & = c_{1} u_{1} + c_{2} u_{2} + c_{5} u_{5} + c_{6} u_{6} ; \\ \tilde{R}_{{ee}} & = c_{3} u_{3} + c_{4} u_{4} + c_{7} u_{5} + c_{8} u_{6} ; \\ \tilde{R}_{{se}} & = c_{9} u_{5} + c_{{10}} u_{6} . \\ \end{aligned}$$

Here, $u_i$ ($i=1,...,6$) indicates the muscle activity of the *i* *th* muscle (see Table 1 for the considered muscles and their indices). The coefficients $c_j$ ($j=1,...,8$) were supposed equal to the ones that linearly related subject joint torques and muscle activities in (2). Whereas, $c_9=\sqrt{c_7 c_5}$ and $c_{10}= \sqrt{c_6c_8}$, with $c_j$ ($j=5,...,8$) obtained from (2).

2 $$\begin{aligned} \tau _{s} & = c_{1} u_{1} - c_{2} u_{2} + c_{5} u_{5} - c_{6} u_{6} ; \\ \tau _{e} & = c_{3} u_{3} - c_{4} u_{4} + c_{7} u_{5} - c_{8} u_{6} . \\ \end{aligned}$$

After computing $\varvec{\tilde{R}}$ with (1), a linear regression between this matrix and the subject joint stiffness matrix ${\varvec{R}}$ provided the coefficients to map $\varvec{\tilde{R}}$ into ${\varvec{R}}$. Thus, knowing the relations mapping ${\textbf{u}}$ in $\varvec{\tilde{R}}$ and $\varvec{\tilde{R}}$ in ${\varvec{R}}$, it was feasible to compute the joint stiffness matrix solely based on the muscle activities. Eventually, the damping matrix ${\textbf{D}}=[{D}_{s}, 0; 0,{D}_{e}]$ was computed from the stiffness matrix by setting $D_s=2\sqrt{|R_{ss}|}$ and $D_e=2\sqrt{|R_{ee}|}$.

#### Kinematic model

In the considered 2-DoF planar task, the arm configuration was completely described by the shoulder and elbow flexion/extension angles. They were computed by exploiting quaternions received from M-IMU sensors placed on the arm and forearm of the participant. First, the model stored the values recorded at the initial time in a T-pose configuration. Then, during the motion, the limb angles (arm and forearm) with respect to the recorded initial T-pose configuration were computed. Eventually, the arm angle determined the shoulder angle, while the elbow angle was computed by subtracting the shoulder angle from the forearm angle.

### Control torque

The arm of the virtual avatar was controlled in the joint space by using a torque control including stiffness and damping components:

3 $$\begin{aligned} \pmb {\tau } = \begin{bmatrix} \tau _{s} \\ \tau _{e} \end{bmatrix} = {\textbf{R}}(\pmb {\theta _{d}} - \pmb {\theta }) + {\textbf{D}}{\pmb {\dot{\theta }}} = \begin{bmatrix} R_{ss} &{} R_{se} \\ R_{se} &{} R_{ee} \end{bmatrix} \left( \begin{bmatrix} \theta _{s,d}\\ \theta _{e,d} \end{bmatrix} - \begin{bmatrix} \theta _{s}\\ \theta _{e} \end{bmatrix} \right) + \begin{bmatrix} D_{s} &{} 0 \\ 0 &{} D_{e} \end{bmatrix} \begin{bmatrix} \dot{\theta }_{s} \\ \dot{\theta }_{e} \end{bmatrix} \end{aligned}$$

In (3) $\tau _{s}$ and $\tau _{e}$ were the shoulder and elbow torques, and $\pmb {\theta _{d}}$ was the vector with the desired joint angles, computed through the *Kinematic Model*. $\pmb {\theta }$ and $\dot{\pmb {\theta }}$ were respectively the vectors containing the actual joint angles and velocities of the virtual arm computed in real-time by the engine of virtual reality. ${\textbf{R}}$ was the joint stiffness matrix and it was assumed to be symmetric, being the anti-symmetric part negligible in the human neuromuscular system^10^. $R_{ss}$ and $R_{ee}$ indicated the diagonal terms (single-joint stiffness), while $R_{se}$ was the off-diagonal component (cross-joint stiffness). Finally, the damping contribution ${\textbf{D}}\,{\pmb {\dot{\theta }}}$ was used to prevent instabilities.

#### Position and impedance controllers

The torque control $\pmb {\tau }$ in (3) was used for both PC and IC. In the case of PC, the stiffness matrix was kept constant, i.e. independent from the actual participant co-contraction, and equal to the average stiffness of the participant in the case of no interaction. Consequently, the participant was allowed to control the virtual arm by only acting on the desired position ($\theta _{j,d}$, with $j \in {s,e}$). On the other hand, during the IC controlled tasks, the impedance coefficients in ${\textbf{R}}$ and ${\textbf{D}}$ were computed through the *Muscle Model*, which allows to map the neuromuscular properties of the participant’s arm into the avatar arm. Hence, the participant gained the capacity to regulate the viscoelasticity of the virtual arm, rather than solely controlling its position.

### Planar reaching task

**Figure 2:**
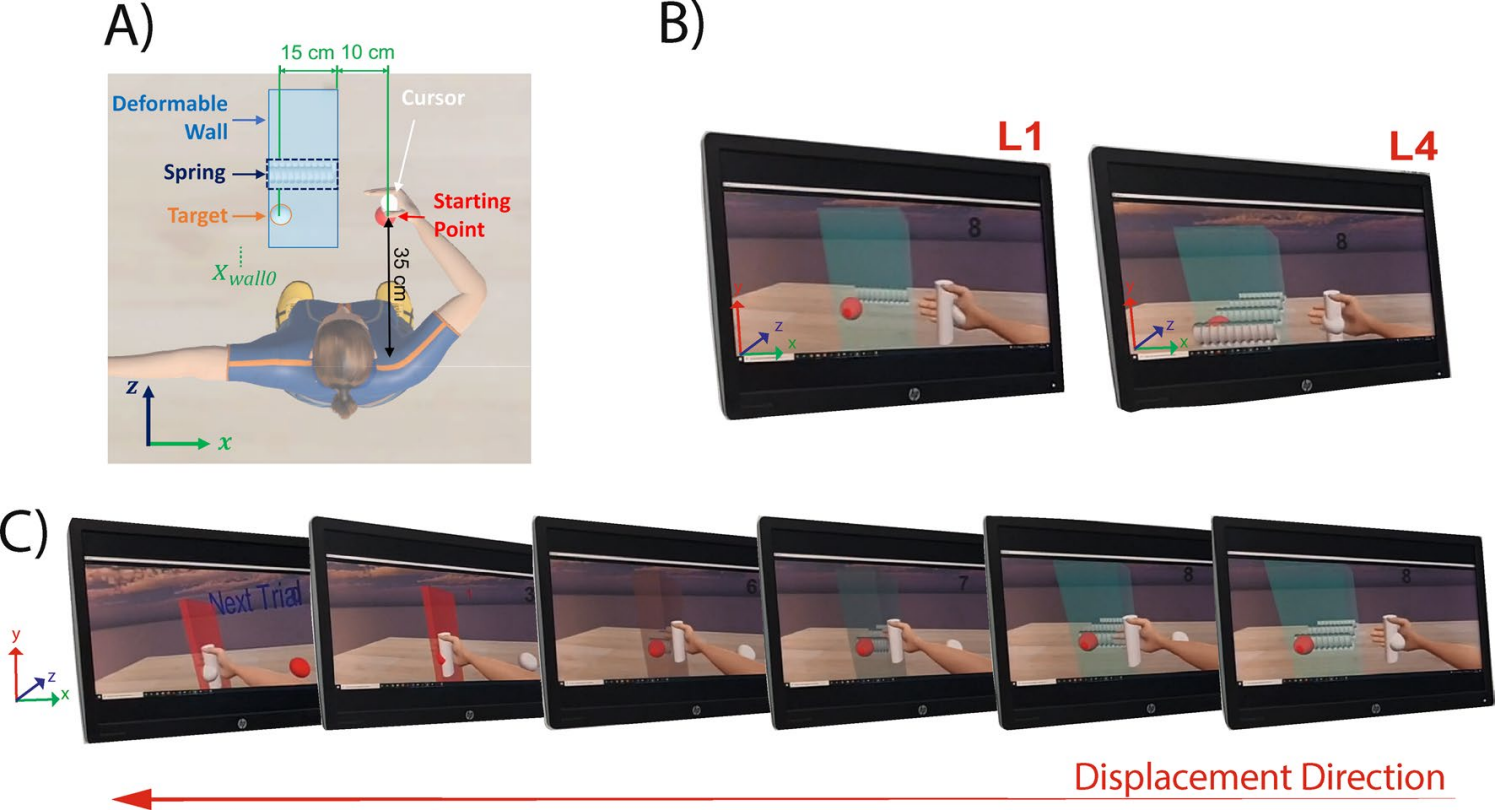
The Virtual Environment used for the experiments. (**A**) General scheme: the starting and the target points are aligned along the *x*-axis and are 25 cm apart. The starting point is aligned with the right shoulder on the *z*-axis with an offset of 35 cm. The elastic wall is represented by a parallelepiped, whose rightmost edge is 10 cm far from the starting point. Inside the wall, one or more springs are shown to provide participants with visual information about the wall stiffness. The cursor placed on the avatar’s hand has to be moved towards the target by the participants. (**B**) First-person perspective seen by the participants for *L*1 and *L*4 wall stiffness levels, indicated with one and four springs, respectively. (**C**) Wall aspect during different moments of the interaction. Six time instants of the same movement (from right to left) are reported to show the changes in the wall aspect when the avatar’s hand interact with it. The last frame shows the end of the trial. The wall is compressed along the *x*-direction. The wall changes colour when compressed, going from light blue to red, which indicates the maximum compression when the cursor is on the target.

Participants were asked to execute a reaching task in virtual reality by controlling the arm of an avatar with the command torque in (3), either in PC and IC conditions. They had to reach a target with a cursor gripped by the avatar’s hand (see Fig. 2) and remain on it for 3 s, after which the task was considered accomplished and the next trial was presented. The maximum overall time acceptable for the completion of the task was set to 10 s. When this time interval was elapsed, the next trial was presented even if the ongoing trial was not successfully completed. The start of the subsequent trial was indicated to the participants by means of a text on the virtual environment, and the countdowns of both 10 and 3 s time intervals were shown during the trial. The starting and the target points became red at the beginning and at the end of the trial, prompting the participants to move towards them. The target was considered reached when the cursor distance from it was less or equal to 2 cm in the virtual reality environment.

The task was performed on a horizontal plane at shoulder height, with movement occurring along the transversal axis (*x*-axis in the task reference system, see Fig. 2A). To simulate real-world interactions with the environment, a virtual elastic wall was introduced between the starting point and the target, inducing the participant to deform it for completing the task. Being the wall modelled as an elastic object, when the avatar pushed it to reach the target, the wall reacted with an elastic force which increases with the displacement of the wall:

4 $$\begin{aligned} F_{w}^x = K_{w}(x_{wall0} - x_{h}). \end{aligned}$$

In (4) $x_{wall0}$ represents the *x* coordinate of the rightmost edge of the wall at the beginning of each trial (i.e. before the interaction), as depicted in Fig. 2A, and $x_{h}$ denotes the *x* coordinate of the avatar hand during the movement. $K_{w}$ indicates the wall stiffness and its value was varied across trials to create five distinct resistance conditions (see "Experimental protocol" section).

The starting point for the avatar hand was aligned with the avatar’s shoulder (same *x* coordinate) with a distance of 35 cm along the *z* direction. The wall and the target were placed respectively 10 cm and 25 cm far from the starting point along *x* direction (see Fig. 2A).

#### Virtual environment

At the beginning and throughout the task, participants can see the starting point, the target and the wall, which delivers the elastic force (4). To simulate different elastic reactions, the wall stiffness $K_w$ was randomly chosen during the task among five values. These conditions were indicated with *Li* ($i = 0,...,4$). The case of null stiffness (*L*0), implying the absence of the wall, was obtained by setting $K_w=0$. To provide the participants with initial visual feedback on the wall stiffness conditions (*L*1, ..., *L*4), a number of springs ranging from 1 to 4 was virtually depicted side by side inside the wall. In Fig. 2 the virtual environment is shown, including a schematic representation of the experimental environment (A) and participant first-person perspective with different wall stiffness values (B).

To increase the perception of the obstacle compression, the wall was visually represented as being pushed by the avatar’s hand during the interaction and its colour was changed from light blue to red as the deformation increased. The change in the wall aspect is shown in Fig. 2C, which represents different time instants of the same movement.

The length of the avatar’s upper arm, forearm and hand were scaled to fit the participant’s ones, thus avoiding any misleading feedback.

### Experimental protocol

Five healthy volunteers took part in the experiments (three females and two males, aged $32.0 \pm 7.5$ years, right-handed) after signing a written informed consent. The experimental protocol was approved by the Ethics Committee of Universitá Campus Bio-Medico di Roma (HUROB protocol) and the research was conducted following the principles of the Declaration of Helsinki.

**Figure 3:**
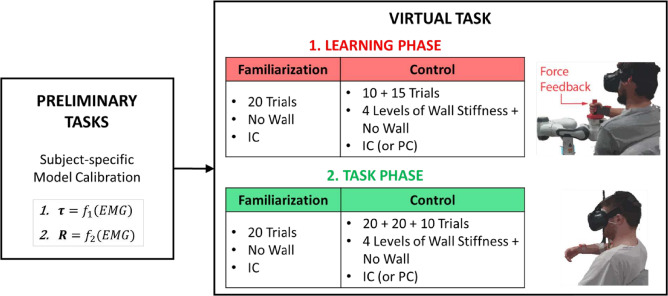
Scheme of the protocol. The overall experiment was conducted in two different sessions, which differed only for the adopted control strategy (PC or IC). At the beginning of each session, participants were required to execute some *Preliminary Tasks* (see "Preliminary tasks" in "Experimental protocol" sections) needed to define the relation between the muscle activation and the joint stiffness matrix. They then performed the *Virtual Task*, in which they had to control the virtual avatar in PC or IC mode. The *Virtual Task* was divided in two phases: (1) a *Learning phase*, during which participants interacted with the robot to perceive an haptic feedback on the interaction force; (2) a *Task phase*, during which participants moved their arm in free space. Both of them are composed by *Familiarization* and *Control* phases, in which the four levels of wall stiffness plus the no-wall condition are pseudo-randomly presented in the two conditions (PC or IC).

Each participant underwent both the IC and PC tasks in two different sessions 1 month apart. The experimental protocol for each session followed the scheme reported in Fig. 3. In both PC and IC, participants were not aware of the adopted control strategy. In three cases, IC was performed before PC, and vice-versa for the other two.

Before starting the experiment we measured the length of the participant’s arm, forearm, and hand. Then, participants were initially required to execute some *Preliminary Tasks* (see "Preliminary tasks" in "Experimental protocol" sections), after which they performed a planar reaching task in virtual reality, teleoperating the arm of the virtual avatar with the control torque explained in "Control torque" section. The virtual task is detailed in "Virtual task" in "Experimental protocol" sections.

#### Preliminary tasks

Preliminary Tasks were required for the computation of the subject-specific parameters of the *Muscular Model*. During most of this phase, participants were asked to grasp the end-effector of a 7-DoFs robotic manipulator (Panda from Franka Emika), configured to work as a 2-DoFs planar robot.

**Figure 4:**
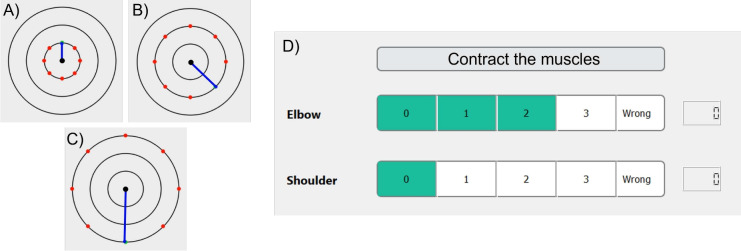
Visual feedback during the preliminary tasks. (**A**–**C**) Visual feedback in the *Force Task* for the three different required force intensities. The blue line indicates the delivered force and the red dots represent the different force intensity-direction required. In each trial, one dot at a time turned green (in random order) to show the user the desired force. (**A**–**C**) show trials relative to desired force intensities of 5 N, 10 N, and 15 N, respectively. (**D**) Visual feedback during the *Perturbation Task*. The two green bars moved along the horizontal direction and showed to the user the co-contraction level for each considered joint, in real-time.

This phase was composed of three tasks:

1. *MVC task*: participants were required to isometrically contract a specific muscle as much as they can against a fixed resistance, represented by an unmovable object. Since we evaluated one muscle per time, the activation of the muscles other than the monitored one is indifferent. This procedure was executed for all the selected muscles (Table 1) to compute the Maximum Voluntary Contraction (MVC) value for each of them.
2. *Force task*: participants exerted a specific level of force on the robotic interface controlled to be highly stiff. The force was applied in 8 directions equally distributed along a circumference. The information on both the direction and the intensity of the desired and actual force was provided to subjects through visual feedback (see Fig. 4A–C). The applied force and the robot displacement were measured through the robotic sensors, while the EMG sensors recorded muscle activities.
3. *Perturbation task*: participants received mechanical perturbations of 6 mm on the hand through the robotic device in 8 directions while recording end-effector displacements and forces, allowing the estimation of joint stiffness^10^. The same protocol was repeated with the participants holding different levels of Co-Contraction for both shoulder and elbow muscles. A visual feedback was provided on Co-Contraction levels to help the participant to keep it constant during robot pertubations (see Fig. 4D). We considered four different Co-Contraction Levels, each one tested four times, resulting in 16 repetitions of this stiffness estimation protocol. As in the previous task, we collected both robot and EMG data.

We detailed each of these tasks in our previous work^32^.

#### Virtual task

The Virtual Task was composed of two phases, during which the subjects had to perform the task in virtual reality:

1. *Learning phase*, to let participants learn the levels of wall stiffness by experiencing a haptic feedback through the robotic interface. To this aim, participants were asked to interact with the robot as in the Preliminary Tasks. In this case, the robot was controlled to be constrained to move only in the x–z plane (see 2A) and to apply a force on the x-axis when the wall was deformed, as defined in (4). During this phase, the avatar was controlled only with the impedance controller to let the participants learn how to modulate their impedance, which would not have happened using the position controller. During this phase, participants had the same visual feedback as the subsequent phase (i.e. *Task phase*), to let them learn the relationship between the visual stimuli and the force intensities.
2. *Task phase*, to test the IC condition and evaluate the participant’s strategies. Participants moved in free space, thus they did not have any haptic feedback on the force applied to the avatar’s hand. Therefore, they could rely only on visual feedback to estimate the wall elastic force.

Each of these phases included two tasks, namely *Familiarization Task* and *Control Task*. In the *Familiarization Task*, consisting of 20 trials, the participants get acquainted with the task without any virtual interaction (no wall was shown during this phase). The trials were performed with or without the robot depending on whether this task was in the *Learning Phase* or in the *Task Phase*, respectively. The values of the stiffness matrix, estimated from the EMG data, were averaged over these trials and then used for the PC condition.

During the *Control Task*, participants experienced the four different wall stiffness values and the no-wall condition. In order to adapt the wall stiffness to the baseline hand stiffness of the participants, the 4 $K_w$ values were computed as a percentage (6%, 12%, 18%, 24%) of the average eigenvalues extracted from the hand stiffness matrices recorded during the *Familiarization Task*. The *Control Task* in the *Learning Phase* and *Task Phase* were composed respectively of 25 and 50 trials, which were organized in blocks interrupted by rest periods of 5 min to avoid participants’ fatigue. The *Learning Phase* had two blocks composed of 10 and 15 trials, whereas the *Task Phase* had 3 blocks of 20, 20 and 10 trials respectively. In each block, the same number of trials for each wall stiffness level (*L*0, ..., *L*4) was presented to participants in a randomized order.

### Experimental setup

**Figure 5:**
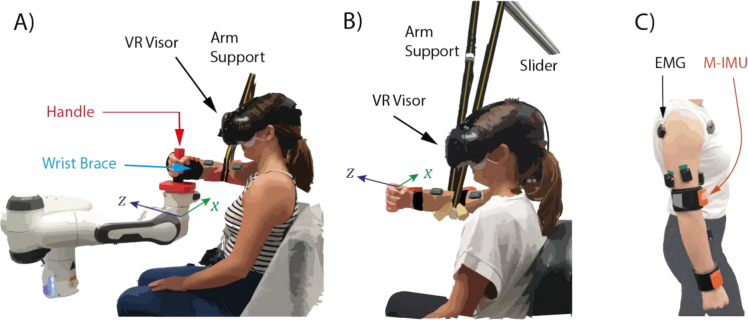
Experimental setup. (**A**) Configuration used for *Preliminary Tasks* and *Learning phase*. Participants grubbed a 3D-printed handle attached to the robot end-effector. The robot is placed on the $x-z$ plane and is used as a haptic interface. Participants wore a wrist brace to guarantee that the task was accomplished only by involving the shoulder and elbow joints (2-DoFs). The arm was supported by a strap to assure the planar motion and minimize muscle activation due to gravity. Six EMG and two M-IMU sensors were placed on participants’ arm, as shown in (**C**), to compute the arm stiffness and position. A Virtual Reality visor was used for providing the visual feedback during the *Learning Phase*. (**B**) Configuration used for the *Task Phase*. The overall setup was almost the same as for the *Learning Phase*. However, in this case, the wrist brace was removed and participants did not interact with the robot. (**C**) Position of EMG and M-IMU sensors used on both phases. Six EMG sensors were placed on two monoarticular muscles and on two biarticular muscles of shoulder and elbow joints. One M-IMU was placed on the forearm and another one on the arm, both close to the elbow and the shoulders to minimize the displacement caused by muscles contraction.

During the *Preliminary Tasks* and the *Learning Phase* participants interacted with a 7-DoF robotic arm (Panda, Franka Emika) configured to operate as a 2-DoF haptic planar robot, as shown in Fig. 5A. On the other hand, during the *Task Phase* they moved the arm in the free space (Fig. 5B).

During the *Preliminary Tasks* participants received visual feedback through a monitor, whereas in the *Virtual Task* they wore the HTC-Vive visor and saw the avatar arm from a first-person perspective. In the experimental phases involving an interaction with the robot, wrist movements were blocked by applying a brace, thus ensuring that the shoulder and elbow joints were solely responsible for the movement. Moreover, an arm support, composed of a strap connected to a slider (Fig. 5B), was used to guarantee the planar motion of the participants’ arm (pure shoulder and elbow flexion/extension) and compensate for arm weight. Six EMG sensors (Trigno$^{\textrm{TM}}$ Wireless System, Delsys) and two M-IMU (MTw$^{\textrm{TM}}$ Awinda, Xsens) placed on participants’ arm, as shown in Fig. 5C, were used to record the activity of muscles listed in Table 1 and kinematic data.

EMG and IMU sensors stream data through wireless communication at 1.11 kHz and 1 kHz, respectively. Then, a TCP/IP communication protocol was developed to send data to a dedicated workstation for real-time elaboration. Data from the robot were recorded at 200 Hz.

The EMG data were real-time filtered with: (a) band-pass filter in the frequency range $20-450$ Hz, (b) notch filter at 50 Hz; (c) low-pass filter with cut-off frequency of 5 Hz. The first and the third filtering were obtained using second-order Butterworth digital filters, while the notch filter was implemented as a second-order IIR notch filter. For each muscle, the filtered EMG data were eventually normalized by the MVC obtained in the *MVC Task* ("Preliminary tasks" in "Experimental protocol" sections). The normalized EMG data were then used in the *Muscle Model* to compute the joint stiffness matrix ${\textbf{R}}$ as function of muscle co-contraction, and used for the real-time avatar control in IC tasks.

The robot was controlled by means of a code developed in C++ within the Qt environment (Qt Creator 4.12.2) and using the Franka Control Interface libraries^35^. The software for providing visual feedback in virtual reality was developed in Unity 3D (2018.1.6f1). The EMG and IMU sensors were acquired through C++ codes developed in Visual Studio and exchanged data with Unity through internal UDP communications. The virtual reality and the sensor software ran on Windows 10, whereas the software controlling the robot ran on Ubuntu 16.04 with a real-time kernel. The two different systems exchanged data through UDP communication.

### Performance metrics

To evaluate the goodness and the reliability of the *Muscle Model* obtained in the *Preliminary Task* we looked at the Pearson’s correlation coefficient $R^2$ for each performed regression. Whereas, the performance of the subjects during the interaction task in PC and IC conditions was evaluated based on the following kinematic and impedance metrics:

- *Joint angles error*: the average of the difference between human and avatar joint angles, calculated as $\Delta q=0.5\sum _{j=s,e} (\theta _{j,d} - \theta _j)$. It represents how well the avatar position replicates the human one. This error can increase for two main interrelated reasons: (1) the first one is that the wall represents an obstacle for the virtual avatar and not for the real participant’s hand who is moving in free space. Therefore, if the force applied by the avatar’s hand on the wall is not enough to move it, the error between the configurations of the two arms increases. (2) The second reason is related to the strategy adopted by the participants to control the avatar. To increase the force with which the avatar pushes against the wall, subjects can augment the joint angle error so as to get the torques in Eq. (3) higher.
- *Task error*: the euclidean distance, computed on the horizontal plane, between the avatar hand and the target. It is an indicator of the attainable precision: the smaller the error, the nearer the hand to the target.
- *Joint stiffness*: the components of the participant’s joint stiffness matrix ${\textbf{R}}$ computed by using the subject-specific *Muscle model*.
- *Hand stiffness*: the components of the hand stiffness matrix ${\textbf{H}}$, computed from the joint stiffness matrix ${\textbf{R}}$ (estimated with the *Muscle model*) as ${\textbf{H}} = {\textbf{J}}^{-T}{\textbf{R}}{\textbf{J}}^{-1}$, where ${\textbf{J}}$ is the arm Jacobian matrix, depending on the participant’s body segments length and joint angles obtained by means of the *Kinematic Model*.
- *Hand Stiffness magnitude and tilt*: the magnitude and the tilt of the participant’s hand stiffness ${\textbf{H}}$. The tilt was computed counterclockwise with respect to the direction of the interaction force (i.e. *x-axis* in Fig. 8). Thus, the smaller the **Hand stiffness tilt**, the more the alignment of the stiffness main axis with the resistance direction. For the sake of simplicity, we will refer to these metrics as *Magnitude* and *Tilt*.
- *Effort*: the sum of the squared motor command^36,37^ of shoulder and elbow, computed by inserting the participant’s joint stiffness in (3) for both PC and IC.

To address how the participants regulated their hand stiffness during the interaction task, we also inspected the hand stiffness ellipses in five different points equally spaced along the trajectory, with the third one corresponding to the contact point with the wall. To compute the stiffness we averaged data belonging to an interval of 0.1 s around each point.

### Data analysis

#### Preliminary tasks

Data from the *Preliminary Tasks* ("Preliminary tasks" in "Experimental protocol" sections) were used to compute the model coefficients associated with each participant, as described in^32^ and briefly summarized in the following:

1. The data saved during the *Force Task* were used for computing the coefficients ${\textbf{c}}$ by a multivariate linear regression between the muscle activity vector ${\textbf{u}}$ and the joint torque vector $\varvec{\tau } = [\tau _s, \ \tau _e] = \mathbf {J^T}{\textbf{F}}$, separately for shoulder and elbow (1). In the computation of $\varvec{\tau }$, ${\textbf{F}} = [F_x; \ F_z]$ was the force recorded at the robot handle and ${\textbf{J}}$ the [2 × 2] Jacobian matrix of the subject’s arm evaluated at the starting position.
2. The data from the *Perturbation Task* were used to estimate the hand stiffness matrix ${\textbf{H}}$, in each of 16 trials, by a linear regression between the variation of the force vector on the plane ($\Delta {\textbf{F}} = [\Delta F_{x};\ \Delta F_{z}]$) and the robot displacement vector ($\Delta {\textbf{x}} = [\Delta {x};\ \Delta {z}]$) (see Fig. 5A for the reference system). The symmetric part of ${\textbf{H}}$, namely ${\textbf{H}}_{sym}$, was extracted and used for the estimation of the joint stiffness matrix ${\textbf{R}} = [R_{ss}, R_{se}; R_{es}, R_{ee}] = {\textbf{J}}^T {\textbf{H}}_{sym} {\textbf{J}}$, with $R_{se} = R_{es}$. In parallel, the vector of muscle activity ${\textbf{u}}$ recorded during the task was employed to compute the matrix of joint pseudo-stiffness $\tilde{{\textbf{R}}}$ using (1) with the coefficients vector ${\textbf{c}}$ obtained from the *Force Task*.
3. Linear regressions between the elements $R_{ss}$, $R_{se}$, $R_{ee}$, of the measured stiffness and the respective ones of the joint pseudo-stiffness matrix ($\tilde{R}_{ss}$, $\tilde{R}_{se}$,$\tilde{R}_{ee}$) was used to estimate the coefficients mapping $\tilde{{\textbf{R}}}$ into ${\textbf{R}}$, thus obtaining the relationship between ${\textbf{u}}$ and ${\textbf{R}}$, used to calculate the joint stiffness matrix in (3) from muscle activation.

#### Control task

The data collected during the *Virtual Task* ("Virtual task" in "Experimental protocol" sections) were elaborated to compare the performance of the subjects in the two experimental conditions PC and IC. The metrics were computed considering data belonging to the 2-second time window characterized by the minimum root-mean-squared variation in the avatar hand’s *x*-coordinate, corresponding to the phase when the hand is in proximity to the target. All the metrics were grouped according to the wall stiffness level and then averaged across all trials with the same conditions. Before running the statistical analysis, the *Effort* was normalised using a logarithmic normalization, whereas the stiffness components and the *Task Error* were not normalised. To evaluate the effect of the control algorithm and wall stiffness on the selected metrics ("Performance metrics section), a two-way Repeated Measures ANOVA or Friedman Test was respectively executed for normal data (*Joint Error* and the logarithm of the *Effort*) and no-normal data (stiffness components and *Task Error*), by using participants as within factor. *Post-hoc* comparisons were executed through a Tukey analysis for normal datasets and with Wilcoxon signed rank test followed by Benjamini & Hochberg procedure for no-normal data.

### Participant consent

Informed consent was obtained from all participants for the publication of identifying information and/or images in online open-access publication.

## Results

### Preliminary tasks

The quality of the three regressions used to estimate subject-specific coefficients of the *Muscle Model* (explained in "Preliminary tasks" in "Data analysis" sections) were evaluated by averaging the Pearson’s correlation coefficient $R^2$ among all the participants. The linear regressions between joint torque and muscle activity for the identification of the coefficients ${\textbf{c}}$ in (1) (item 1 of "Preliminary tasks" in "Data analysis" sections) had the $R^2$ values in Table 2. The values of $R^2$ obtained for the estimation of the hand stiffness ${\textbf{H}}$ (item 2 of "Preliminary tasks" in "Data analysis" sections) are shown in Table 3. Finally, the regressions between the components of the matrix of joint pseudo-stiffness $\mathbf {\tilde{R}}$ and the matrix of the measured stiffness ${\textbf{R}}$ (item 3 of "Preliminary tasks" in "Data analysis" sections) were characterized by the $R^2$ values shown in Table 4. Notably, these $R^2$ values reflected the similarity between the estimated and actual stiffness, thus providing an indication of the quality and reliability of the overall model.

**Table 2:** The Pearson’s $R^2$ values for the regression joint torque $\varvec{\tau }$ and muscle activity ${\textbf{u}}$ in (1).

Joint	R^2^
Shoulder	$0.81 \pm 0.08$
Elbow	$0.86 \pm 0.06$

**Table 3:** The Pearson’s $R^2$ values for the regression between displacement vector and the variation of $F_x$ or $F_z$, executed for each Co-Contraction Level.

Co-Contraction	R^2^	
Level	$\Delta F_x$ versus $\Delta {\text{x}}$	$\Delta F_z$ versus $\Delta {\text{x}}$
0: $0-4$%	$0.64 \pm 0.12$	$0.84 \pm 0.10$
1: $4-8$%	$0.78 \pm 0.14$	$0.83 \pm 0.09$
2: $8-12$%	$0.77 \pm 0.12$	$0.77 \pm 0.11$
3: $12-16$%	$0.72 \pm 0.23$	$0.69 \pm 0.28$

**Table 4:** The Pearson’s $R^2$ values for the regression between the components of the joint pseudo-stiffness matrix $\mathbf {\tilde{R}}$ and the measured stiffness matrix ${\textbf{R}}$.

Matrix components	R^2^
$R_{ss}$ versus $\tilde{R}_{ss}$	$0.64 \pm 0.12$
$R_{se}$ versus $\tilde{R}_{se}$	$0.85 \pm 0.10$
$R_{ee}$ versus $\tilde{R}_{ee}$	$0.66 \pm 0.12$

### Virtual task

**Figure 6:**
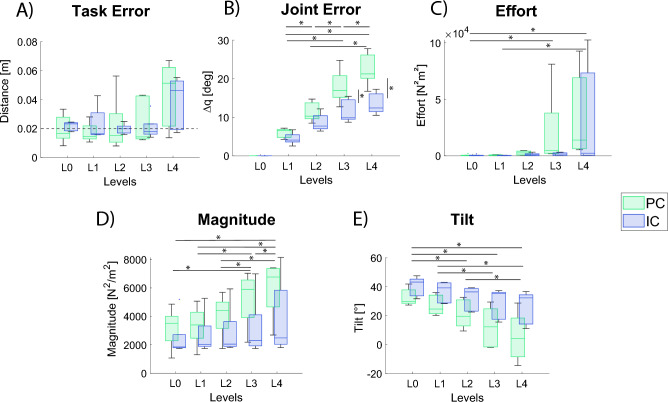
Performance metrics. Each box plot shows the performance trend depending on the wall stiffness condition (*Li*, $i = 0,...,4$). PC and IC are indicated with light and dark blue, respectively. The distributions are displayed considering the mean value for each participant among all the trials with the same wall stiffness. (**A**) *Task Error*: Euclidean distance in the virtual reality between the cursor and the target (**B**) *Joint Error*: sum of the difference between the avatar and the participant joints’ angles (**C**) *Effort*: sum of the squared command torque. (**D**) *Magnitude*: magnitude of the hand stiffness ellipse. (**E**) *Tilt*: the tilt of the hand stiffness ellipse with respect to the direction of the elastic force. The tilt angle is computed between the main axis of the hand stiffness ellipses and the axis of the interaction force. Each box plot shows the median, the upper and lower quartile. The whiskers indicate the minimum and maximum values that are not outliers * indicates a statistically significant difference among groups when using Tukey’s honest significance test as post-hoc (all metrics except for *Magnitude*). * indicates *p*<0.05 in the Wilcoxon signed rank test corrected with Benjamini and Hochberg procedure (used for the *Magnitude*).

The evaluated performance metrics with respect to the wall stiffness level are shown in Fig. 6, for both PC and IC.

The task error (Fig. 6A) shows that participants completed the task with an average error of around 2 cm for all wall stiffness levels, except for the last one in which the error is considerably higher, i.e. about 5 cm. Hence, since the target was considered reached when the hand position fell within a tolerance distance of 2 cm, all the tasks with stiffness values up to L3 were successfully completed.

Different behaviours between PC and IC could be observed from the *Joint Error* (Fig. 6B), especially in the case of high values of wall stiffness (L3 and L4 conditions). Without the wall (L0 condition), the kinematic pattern of the participants’ arm was the same as the avatar for both PC and IC. When the virtual wall was introduced, participants compensated for its resistance by moving their own arm beyond the one of the avatar. The same trend over the stiffness levels could be observed for both controllers: the higher the wall stiffness, the higher the joint angles error ($p<0.001$ and $L1<L2<L3<L4$, in this case, being the error very near to 0 in *L*0, it was not considered in the statistical analysis). However, joint angles error increased more in PC condition than in IC. Statistical analysis revealed a significant effect of the interaction between stiffness levels and controllers ($p<0.001$) and post-hoc comparisons showed a significant lower error for IC in the case of *L*3 and *L*4 conditions with respect to PC.

A similar trend could be also observed for the *Effort* and the *Magnitude* (Fig. 6C and D), which increased with wall stiffness levels ($p<0.001$) and was on average higher for PC than for IC, although not significantly different among the two conditions.

Interestingly, a slightly different behaviour (not statistically significant) between PC and IC can be observed looking at the *Tilt* of the hand stiffness ellipses when the avatar hand is in the proximity of the target. Although in both conditions the median value of the tilt decreased when the wall stiffness increased, the slope of this decrement is less in IC than in PC, where the median tilt value went from about 30° in L0 to about 0° in L4. The mentioned trend of both *Magnitude* and *Tilt* can be observed by looking at the stiffness ellipses of both the controllers in Point 5 of the Fig. 8.

**Figure 7:**
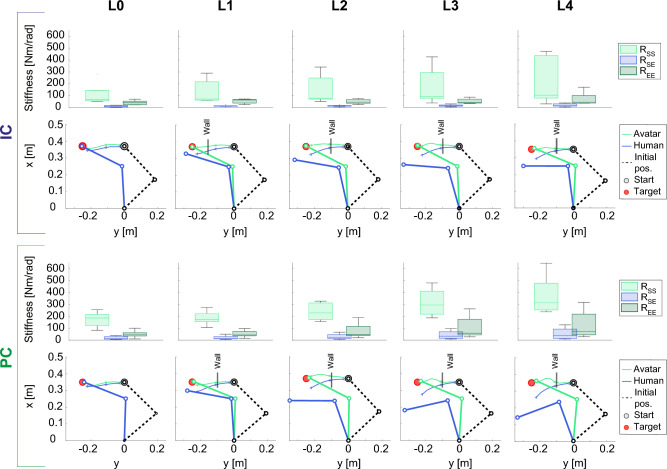
Overall view on the control strategies for IC (top) and PC (bottom). The stiffness management is shown in the first row of each condition, displaying the three components of the joint stiffness matrix ($R_{ss}, R_{se}, R_{ee}$). Each box plot describes the distribution among participants for the specific wall stiffness level (*Li*, $i = 0,...,4$). In the lower row, position management is schematically represented showing the avatar’s and participant’s arm configuration at the beginning and at the end of the task. The initial positions are coincident for all the wall stiffness levels, therefore only one line is visible. In addition, the trajectories executed by the avatar’s and participant’s hand, going from the start (grey dot) to the target (red dot), are displayed in light blue and dark blue, respectively. The arm configurations are drawn by averaging the desired and real position of elbow and hand among all the participants and considering the shoulder fixed in the point with coordinates $x=0$ and $y=0$.

A global representation of the control strategy, considering both the position and the stiffness components, is shown in Figure 7. For each controller, the components of the joint stiffness matrices for each *Li* level are shown in the upper row, while the bottom row represents the arm configurations and hand trajectories of the participant (dark blue) and of the avatar (light blue). The trajectories were obtained by averaging data among participants at 8 evenly spaced points within the range $[-0.22, -0.028]$ m. We analyzed this reduced spatial interval, i.e. not the full range from the starting point ($x=0$ m) to the target ($x=-0.25$ m), to remove the initial and final parts of the trajectories which are usually affected by transitory adjustment movements. The initial and final arm configurations were computed by averaging the joint angles of the hand and elbow at the beginning and end of the trial, for both the avatar and participant’s data. The box plots showed an increase in the arm stiffness components with the wall stiffness level ($L_i$) in both PC and IC conditions, with a statistically significant difference among levels in $R_{se}$ ($p=0.012$ with $L0=L1=L2<L3<L4$). In addition, all the components of the joint stiffness matrix were on average higher in PC than IC, although statistically different only in the case of $R_{ss}$ ($p=0.0068$ with $R_{ss,PC} > R_{ss, IC}$). As expected by the *Joint Errors* trend (Fig. 6B), the avatar and human arm configurations were nearly identical without the wall. Then, the disparity increased for wall stiffness levels beyond *L*0, with the elbow angle generally more flexed in PC than in IC.

**Figure 8:**
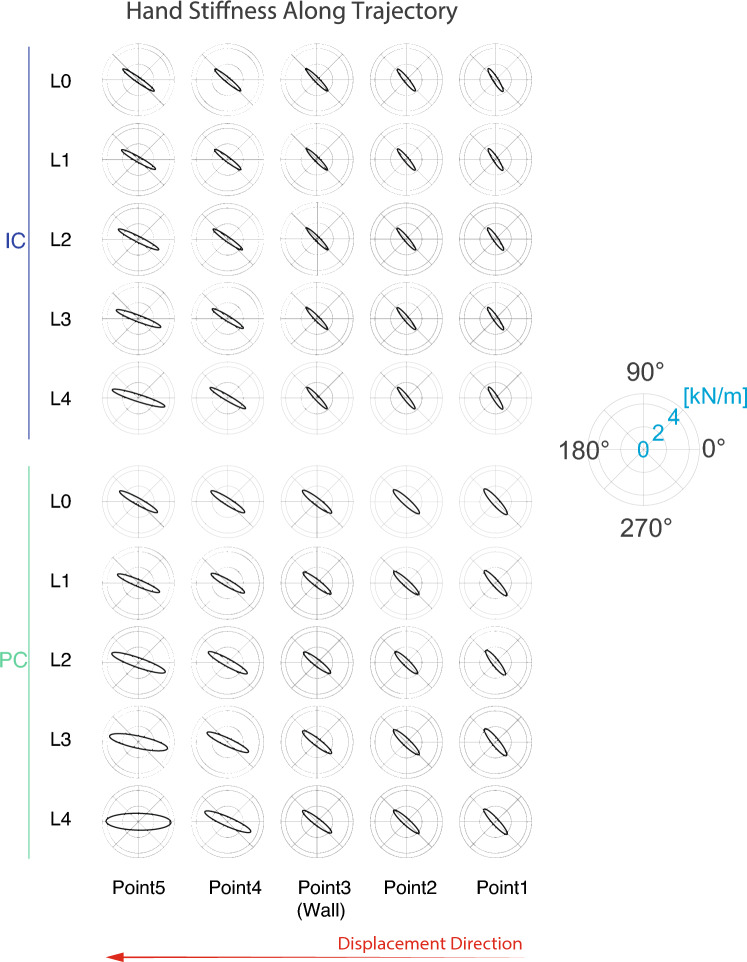
Hand stiffness ellipses in five points along the trajectory for each wall stiffness level *Li* and controller. Going from right to left, each row describes the trend of hand stiffness along the trajectory for a specific level of wall stiffness. Point3 coincides with the contact point with the wall; the other points are equally spaced.

Finally, the hand stiffness ellipse observed in five different points along the task trajectory (*x* direction) is shown in Fig. 8. In agreement with the trend shown in Figs. 7 and in 6, in the PC condition the magnitude of the hand stiffness ellipse was higher than in the IC condition. The shape and the magnitude of the ellipse before the wall (up to Point3) remained unaltered for all the wall stiffness levels within the same controller, and changed after the wall (Point4 and Point5). This change was more evident for high values of wall stiffness (*L*4 and *L*5) with Point5 having a higher magnitude and a lower tilt angle than Point4. In both PC and IC, when considering the same point (Point4 or Point5), the higher the wall stiffness level, the higher the stiffness magnitude. In addition, in both types of controllers, the tilting caused by the increase of wall stiffness tended to align the major axis in the direction of displacement (i.e. against the obstacle), corresponding to the direction of the elastic force arising from the interaction with the wall. Such tilting was more noticeable in PC than in IC, being the stiffness ellipse completely horizontal in the former case.

## Discussion

Starting from the better performance of a tele-impedance controller based on human real-time data (position and impedance) with respect to a position controller^21^, we tested a similar approach based on different estimation algorithms in a virtual reaching task. Ajoudani et al.^9,21^ controlled the robot in operational space with the hand position reconstructed by means of an optoelectronic system and the hand stiffness estimated based on muscular activity responsible for stiffness modulation. Conversely, we implemented the controller in the joint space, thus directly mapping on the avatar the estimated human joint angles and impedance (using a linear model between stiffness and muscle activation^12^). By focusing on joint stiffness rather than task space stiffness, we could avoid the high dependency of the computed stiffness on the arm configuration^38^. For this reason, we assumed that the variation of joint stiffness with respect to the arm pose was negligible within the task workspace, thus considering the model coefficient constant within each subject. By computing both joint angles and stiffness through portable wearable sensors (IMU and EMG sensors), our algorithm allows the exploitation of the tele-impedance control in joint space in unstructured environments, as it does not need a camera-based tracking system.

Although some tele-impedance solutions using EMG and M-IMU sensors have been proposed in previous studies^31,33,34^, our setup differs from them for not involving a master robotic system^33^, and for the capability of our controller to map the joint user’s impedance on the slave^31,34^. Laghi et al.^33^ exploited the tele-impedance paradigm within a two-channel bilateral architecture requiring two robotic systems used as master and slave. Consequently, this approach, differently from ours, involves a bilateral system rather than a classic tele-impedance control, for which both the configuration and impedance of the user’s arm are mapped on the slave system without involving a master robot^22^. In Laghi’s paper, the configuration of the arm is estimated using both M-IMU sensor and the robotic master, thus the proposed tele-impedance algorithm could not be extended to a portable control setup. Moreover, while we tested our algorithm in an interaction task requiring the appliance of a target force, they exploited their strategy based on M-IMU in a simple obstacle detection task. Indeed, they changed the setup and substituted the M-IMU sensors with an optoelectronic system when considering a more complex interaction task. On the other hand, the approach proposed by Lian et al.^34^ meets the portability requirement by using only M-IMU sensors for mapping the user’s configuration onto the robotic system. However, their control modulated the impedance of the robotic system based on the robot’s position in the workspace, not directly estimating the user’s arm impedance. In contrast, our tele-impedance controller aimed to map both the user’s impedance and position into the remote system, providing it with human-like control strategy, following the tele-impedance idea proposed by Ajoudani et al.^21,22^. Wu et al.^31^ proposed a portable tele-impedance solution that mapped the pose and stiffness of the user’s hand onto the slave end-effector. Our approach focused on mapping joint impedance, instead of hand stiffness. This mitigated the high dependence between stiffness and workspace, and allowed us to extend control into an impedance framework. Summing up, the main innovations of our tele-impedance control are related to the combination of the advantages of portability and direct joint impedance mapping for improved control.

We assessed the performance of only five participants due to the proof-of-concept nature of this study. The results showed that the proposed tele-impedance controller allowed participants to fulfill the 2-DoFs planar reaching task for all wall stiffness levels, except for the highest one (*L*4). The participants’ failure in reaching the task goal for *L*4, in both PC and IC conditions, can be reasonably due to the high value of the wall stiffness. Nonetheless, we showed that having control over the system’s impedance can have some benefits in a task where a given target interaction force has to be achieved, and that, also in the absence of explicit force feedback, it is possible to implement a tele-impedance control using solely wearable sensors, so to pave the way for its use in out-of-the-lab applications.

Despite the *Task Error* being similar for PC and IC, the two conditions differed for the control strategies adopted by the participants to complete the task, as shown by the *Joint Error* and *Stiffness* regulation.

Firstly, the high difference between human and avatar kinematics in PC could be attributed to the lack of impedance modulation in the control torque (3) for this condition, which conversely was present in IC. Indeed, since IC control torque allows the regulation of both the desired motion and the impedance, participants could independently modulate the kinematics of movement and the interaction behaviour of the avatar. On the contrary, as in the PC the impedance was constant, participants could only deal with the external constraint by changing the desired position. Hence, in PC condition participants had to move further their arm with respect to the target position when they needed to counteract the wall resistance, especially for high values of wall stiffness. Therefore, since increasing the discrepancy was the only means for participants to accomplish the task in PC, a higher disparity between the avatar’s and the participant’s arm configurations was observed in PC than in IC, especially for the higher wall stiffness levels. This detected difference in posture between the user and the remote system could potentially lead to reduced intuitiveness during the control.

Secondly, we found a different management of the limb stiffness, estimated by the Muscle Model, between the two controllers. Even though in both PC and IC the components of the joint stiffness matrix and the stiffness magnitude increased with the wall stiffness level, the stiffness in PC was on average higher than in IC, in terms of both single components and magnitude. Another difference between the two controllers was observed in the tilting of the stiffness ellipses across wall stiffness levels. In both conditions, the stiffness ellipses tended to align their main axis with the *x*-axis (i.e. the direction of the wall generated force) when the wall stiffness increased, as depicted by a decrease of the tilt angle observed in Figs. 6 and 8. However, in PC we noticed a lower tilt value (i.e. on average almost zero), so that the ellipses became almost horizontal for the highest level of wall stiffness (*L*4). The difference between the stiffness regulation in the two conditions can be attributed to the lack of changes in the avatar’s behavior following stiffness modulation in the case of PC. Indeed, to compensate for the avatar’s missed adaptation, participants tended to increase the stiffness beyond the necessary value, in particular along the direction needed to counteract the wall resistance. It is also worth noticing that part of this effect can be due to the need of participant to flex their shoulder more in PC than in IC, thus having to fight the intrinsic visco-elastic effects of their own antagonistic musculature under tension. However, except for the last point, the overall higher stiffness value could be explained by a difference in the human control between the two strategies which caused higher stiffness magnitude in PC. Therefore, it could be argued that PC required more effort than IC.

Hence, with this study, we confirmed the better performance of IC with respect to PC^9,21,29^, and we proposed a new teleimpedance solution , requiring only the use of portable and wearable technologies. Our solution is, therefore, suitable to be applied in a wider range of applications implying unstructured environments, overcoming the limitations of previous solutions^9,21,29^.

From the comparison between IC and PC, we also gained useful insights into the human motor strategy during teleoperation. The change in stiffness orientation suggested that, also during teleoperation tasks, humans tend to directionally tune their arm stiffness (i.e. increasing the stiffness selectively along the perturbed direction) as they usually do in controlling their own arm^18,39^. This is done regardless of the presence of haptic feedback and also when this change does not affect the tele-controlled arm. Thus, we confirmed the important role of impedance regulation in motor control during interaction tasks^10,15,18,39^.

The presented results suggest the feasibility of using our control approach for teleoperation in joint space based on wearable sensors, opening the way to its applications in unstructured environments. Even if preliminary tested in a virtual environment, this work constitutes a first validation step towards the application of this approach to real remote robotic systems. However, we recognize some limitations in the study that may be improved in future works. First of all, the task designed could be improved to: (1) avoid reaching the user’s joint limits to prevent stiffness increases related to muscle visco-elasticity during the PC control, and (2) include some instabilities to gain better insight into the difference in performance between the two controllers. Additionally, the large number of parameters in the muscle model could be reduced to shorten the calibration phase. It is also important to note that although the current setup is not portable in the calibration phase, potential workarounds using portable devices rather than a robotic system could address this issue. For instance, load cells and M-IMU sensors could be exploited for measuring interaction forces and estimating arm movements. Using such a setup, the user can execute an isometric force task against a fixed object for the *Force Task*, and the experimenter can perturb the user’s hand for the *Perturbation Task*. Finally, the absence of force feedback during tasks could impact participants’ performance by preventing them from better feeling the remote environment by merging the visual and the touch sensing^40,41^.

Further investigations can be devoted to improve the identified limitations. Firstly, we aim to test the utility of additional feedback within the proposed tele-impedance system. In this case, attention will be given to using technologies that would not impact the portability of the system, such as vibrotactile or electrotactile technologies. As stated before, these additional feedback could enhance the information on the interaction force and the overall environment, especially if targeted applications entail environment instabilities. Direct feedback on interaction forces may enable participants to modulate their stiffness more intuitively. Finally, to allow for a complete portability of the system, future studies will focus on defining and testing a calibration phase setup that includes only portable devices.

## Data Availability

The datasets generated during the current study are available in the *Mendeley Data* online repository.

## References

[1] Chen, H., Huang, P. & Liu, Z. Mode switching-based symmetric predictive control mechanism for networked teleoperation space robot system. *IEEE/ASME Trans. Mechatron.* **24**, 2706–2717 (2019). 10.1109/TMECH.2019.2946197

[2] Diftler, M. A. *et al.* Robonaut 2-the first humanoid robot in space. In *2011 IEEE International Conference on Robotics and Automation* 2178–2183 (IEEE, 2011).

[3] Sivčev, S., Coleman, J., Omerdić, E., Dooly, G. & Toal, D. Underwater manipulators: A review. *Ocean Eng.* **163**, 431–450 (2018). 10.1016/j.oceaneng.2018.06.018

[4] Panzirsch, M., Sierotowicz, M., Prakash, R., Singh, H. & Ott, C. Deflection-domain passivity control of variable stiffnesses based on potential energy reference. *IEEE Robot. Autom. Lett.* **7**, 4440–4447 (2022). 10.1109/LRA.2022.3147566

[5] Panzirsch, M., Singh, H., Sierotowicz, M. & Dietrich, A. Extension of the deflection-domain passivity approach for variable stiffnesses to so (3). *IEEE Robot. Autom. Lett.* **9**, 2925–2932 (2024). 10.1109/LRA.2024.3358584

[6] Ozturkcan, S. & Merdin-Uygur, E. Humanoid service robots: The future of healthcare?. *J. Inform. Technol. Teach. Cases* **12**, 163–169 (2022). 10.1177/20438869211003905

[7] Hung, L. *et al.* Telepresence robots in long-term care settings in British Columbia during the covid-19 pandemic: Analyzing the experiences of residents and family members. *Gerontol. Geriatr. Med.* **9**, 23337214231166210 (2023). 37033088 10.1177/23337214231166208 PMC10076606

[8] Noccaro, A. *et al.* A teleoperated control approach for anthropomorphic manipulator using magneto-inertial sensors. In *2017 26th IEEE International Symposium on Robot and Human Interactive Communication (RO-MAN)*, 156–161 (IEEE, 2017). 10.1109/ROMAN.2017.8172295 PMC6445357 30949293

[9] Ajoudani, A., Tsagarakis, N. G. & Bicchi, A. Tele-impedance: Preliminary results on measuring and replicating human arm impedance in tele operated robots. In *Robotics and Biomimetics (ROBIO), 2011 IEEE International Conference on* 216–222 (IEEE, 2011).

[10] Mussa-Ivaldi, F. A., Hogan, N. & Bizzi, E. Neural, mechanical, and geometric factors subserving arm posture in humans. *J. Neurosci.* **5**, 2732–2743 (1985). 4045550 10.1523/JNEUROSCI.05-10-02732.1985 PMC6565149

[11] Gomi, H. & Osu, R. Task-dependent viscoelasticity of human multijoint arm and its spatial characteristics for interaction with environments. *J. Neurosci.* **18**, 8965–8978 (1998). 9787002 10.1523/JNEUROSCI.18-21-08965.1998 PMC6793558

[12] Osu, R. & Gomi, H. Multijoint muscle regulation mechanisms examined by measured human arm stiffness and EMG signals. *J. Neurophysiol.* **81**, 1458–1468 (1999). 10200182 10.1152/jn.1999.81.4.1458

[13] Franklin, D. W., Osu, R., Burdet, E., Kawato, M. & Milner, T. E. Adaptation to stable and unstable dynamics achieved by combined impedance control and inverse dynamics model. *J. Neurophysiol.* **90**, 3270–3282 (2003). 14615432 10.1152/jn.01112.2002

[14] Burdet, E. *et al.* Stability and motor adaptation in human arm movements. *Biol. Cybern.* **94**, 20–32 (2006). 16283374 10.1007/s00422-005-0025-9

[15] Shadmehr, R. & Mussa-Ivaldi, F. A. Adaptive representation of dynamics during learning of a motor task. *J. Neurosci.* **14**, 3208–3224 (1994). 8182467 10.1523/JNEUROSCI.14-05-03208.1994 PMC6577492

[16] Gribble, P. L., Mullin, L. I., Cothros, N. & Mattar, A. Role of cocontraction in arm movement accuracy. *J. Neurophysiol.* **89**, 2396–2405 (2003). 12611935 10.1152/jn.01020.2002

[17] Heald, J. B., Franklin, D. W. & Wolpert, D. M. Increasing muscle co-contraction speeds up internal model acquisition during dynamic motor learning. *Sci. Rep.* **8**, 16355 (2018). 30397273 10.1038/s41598-018-34737-5 PMC6218508

[18] Burdet, E., Osu, R., Franklin, D. W., Milner, T. E. & Kawato, M. The central nervous system stabilizes unstable dynamics by learning optimal impedance. *Nature* **414**, 446–449 (2001). 11719805 10.1038/35106566

[19] Tagliamonte, N. L., Accoto, D. & Guglielmelli, E. Rendering viscoelasticity with series elastic actuators using cascade control. In *2014 IEEE International Conference on Robotics and Automation (ICRA)* 2424–2429 (IEEE, 2014).

[20] Accoto, D. *et al.* pvej: A modular passive viscoelastic joint for assistive wearable robots. In *2012 IEEE International Conference on Robotics and Automation* 3361–3366 (IEEE, 2012). 10.1109/ICORR.2011.5975356 22275560

[21] Ajoudani, A., Tsagarakis, N. & Bicchi, A. Tele-impedance: Teleoperation with impedance regulation using a body-machine interface. *Int. J. Robot. Res.* **31**, 1642–1656 (2012). 10.1177/0278364912464668

[22] Ajoudani, A. Teleimpedance: Teleoperation with impedance regulation using a body-machine interface. In *Transferring Human Impedance Regulation Skills to Robots* (ed. Ajoudani, A.) 19–31 (Springer, 2016).

[23] Hogan, N. Adaptive control of mechanical impedance by coactivation of antagonist muscles. *IEEE Trans. Autom. Control* **29**, 681–690 (1984). 10.1109/TAC.1984.1103644

[24] Yang, C. *et al.* Human-like adaptation of force and impedance in stable and unstable interactions. *IEEE Trans. Rob.* **27**, 918–930 (2011). 10.1109/TRO.2011.2158251

[25] Shao, Q., Bassett, D. N., Manal, K. & Buchanan, T. S. An EMG-driven model to estimate muscle forces and joint moments in stroke patients. *Comput. Biol. Med.* **39**, 1083–1088 (2009). 19818436 10.1016/j.compbiomed.2009.09.002 PMC2784179

[26] Ambrósio, J., Quental, C., Pilarczyk, B., Folgado, J. & Monteiro, J. Multibody biomechanical models of the upper limb. *Procedia IUTAM* **2**, 4–17 (2011). 10.1016/j.piutam.2011.04.002

[27] Sartori, M., Maculan, M., Pizzolato, C., Reggiani, M. & Farina, D. Modeling and simulating the neuromuscular mechanisms regulating ankle and knee joint stiffness during human locomotion. *J. Neurophysiol.* **114**, 2509–2527 (2015). 26245321 10.1152/jn.00989.2014 PMC4620138

[28] Osu, R. *et al.* Short-and long-term changes in joint co-contraction associated with motor learning as revealed from surface EMG. *J. Neurophysiol.* **88**, 991–1004 (2002). 12163548 10.1152/jn.2002.88.2.991

[29] Ajoudani, A., Fang, C., Tsagarakis, N. G. & Bicchi, A. A reduced-complexity description of arm endpoint stiffness with applications to teleimpedance control. In *2015 IEEE/RSJ International Conference on Intelligent Robots and Systems (IROS)* 1017–1023 (IEEE, 2015).

[30] Fang, C., Ajoudani, A., Bicchi, A. & Tsagarakis, N. G. Online model based estimation of complete joint stiffness of human arm. *IEEE Robot. Autom. Lett.* **3**, 84–91 (2017). 10.1109/LRA.2017.2731524

[31] Wu, Y. *et al.* A teleoperation interface for loco-manipulation control of mobile collaborative robotic assistant. *IEEE Robot. Autom. Lett.* **4**, 3593–3600 (2019). 10.1109/LRA.2019.2928757

[32] Buscaglione, S. *et al.* Tele-impedance control approach using wearable sensors. In *2022 44th Annual International Conference of the IEEE Engineering in Medicine & Biology Society (EMBC)* 2870–2873 (IEEE, 2022). 10.1109/EMBC48229.2022.9871736 36086036

[33] Laghi, M., Ajoudani, A., Catalano, M. G. & Bicchi, A. Unifying bilateral teleoperation and tele-impedance for enhanced user experience. *Int. J. Robot. Res.* **39**, 514–539 (2020). 10.1177/0278364919891773

[34] Lian, S. *et al.* Improving teleoperation of high degree-of-freedom robots with a dual-mode system and variable admittance controller using imu-based tracking. In *2023 IEEE 13th International Conference on CYBER Technology in Automation, Control, and Intelligent Systems (CYBER)* 1293–1298 (IEEE, 2023).

[35] Libfranka: C++ library for franka emika research robots. https://frankaemika.github.io/libfranka/ (2022).

[36] Diedrichsen, J., Shadmehr, R. & Ivry, R. B. The coordination of movement: Optimal feedback control and beyond. *Trends Cogn. Sci.* **14**, 31–39 (2010). 20005767 10.1016/j.tics.2009.11.004 PMC4350769

[37] Todorov, E. & Jordan, M. I. Optimal feedback control as a theory of motor coordination. *Nat. Neurosci.* **5**, 1226–1235 (2002). 12404008 10.1038/nn963

[38] Shadmehr, R. Control of equilibrium position and stiffness through postural modules. *J. Mot. Behav.* **25**, 228–241 (1993). 12581992 10.1080/00222895.1993.9942052

[39] Franklin, D. W. *et al.* Endpoint stiffness of the arm is directionally tuned to instability in the environment. *J. Neurosci.* **27**, 7705–7716 (2007). 17634365 10.1523/JNEUROSCI.0968-07.2007 PMC6672883

[40] Held, R. & Durlach, N. Telepresence. *Presence: Teleoperators and Virtual Environments* **1**(1), 109–112 (1992) 10.1162/pres.1992.1.1.109

[41] Preusche, C. Telerobotics. In *Springer Handbook of Robotics* (eds Siciliano, B. *et al.*) 741–757 (Springer, 2008).

